# Invasive Papillary Carcinoma of the Breast: Clinicopathological Features and Hormone Receptor Profile

**DOI:** 10.7759/cureus.13480

**Published:** 2021-02-22

**Authors:** Atif A Hashmi, Shahzeb Munawar, Naumana Rehman, Omer Ahmed, Sabeeh Islam, Ishaq Azeem Asghar, Anoshia Afzal, Muhammad Irfan, Farozaan Shamail, Syed J Ali

**Affiliations:** 1 Pathology, Liaquat National Hospital and Medical College, Karachi, PAK; 2 Internal Medicine, Liaquat College of Medicine and Dentistry, Karachi, PAK; 3 Pathology, Khyber Medical University, Peshawar, PAK; 4 Internal Medicine, Liaquat National Hospital and Medical College, Karachi, PAK; 5 Internal Medicine, St. Vincent Health Center, Buffalo, USA; 6 Internal Medicine, Faisalabad Medical University, Faisalabad, PAK; 7 Pathology, Ascension St. John Hospital, Detroit, USA; 8 Pathology, University of Oklahoma Health Sciences Center, Oklahoma City, USA; 9 Statistics, Liaquat National Hospital and Medical College, Karachi, PAK; 10 Pathology, Ziauddin University, Karachi, PAK; 11 Pathology, Dow University of Health Sciences, Karachi, PAK

**Keywords:** invasive papillary carcinoma, invasive ductal carcinoma, breast cancer, estrogen receptor, progesterone receptor, human epidermal growth factor receptor 2, ki67, papillary lesions

## Abstract

Introduction

Papillary neoplasms are a heterogeneous group of breast lesions, ranging from benign to in situ and invasive malignant tumors. The term invasive papillary carcinoma (IPC) is reserved for rare invasive breast tumors showing greater than 90% papillary morphology. The clinical, epidemiological and pathological characteristics of IPC are not widely described in the existing literature; therefore, in this study, we evaluated the clinicopathological features and biomarker profile of IPC and compared it with invasive ductal carcinoma (IDC) diagnosed in the same study duration.

Methods

A retrospective study was conducted in the Department of Histopathology, Liaquat National Hospital and Medical College, from January 2013 to December 2020. During the study period, 44 cases of IPC and 1,268 cases of IDC were diagnosed. Slides and blocks of all cases were retrieved and histopathological diagnosis was reviewed. Estrogen receptor (ER), progesterone receptor (PR), human epidermal growth factor receptor 2 (HER2/neu), and Ki67 immunohistochemical (IHC) stains were applied on representative tissue blocks.

Results

The mean age of the patients with IPC was 58.77±8.38 years, and the mean Ki67 index was 19.95±21.12%. The mean tumor size was 32.41±17.39 mm, and most tumors (59.1%) were tumor (T)-stage T2. Axillary metastasis was present in 13.6% cases, and 86.4% cases had nodal (N)-stage N0. ER and PR expression was noted in 72.7% cases, and HER2/neu positivity was seen in 13.6% cases. IPC cases had a higher mean age than IDC. Conversely, IPC had a lower mean Ki67 index than IDC. Similarly, IPC cases were found to have a lower frequency of axillary metastasis than IDC. IPC was noted to have a lower frequency of T3-stage and lymphovascular invasion than IDC. A higher expression of PR and lower frequency of HER2/neu expression was noted in IPC than IDC.

Conclusion

IPC is a rare malignant papillary breast tumor with a wide differential diagnosis and therefore poses a significant diagnostic challenge. We found that IPC had a favorable pathological profile than IDC, in terms of T-stage, Ki67 index, axillary metastasis, PR and HER2/neu expression.

## Introduction

Papillary neoplasms are a heterogeneous group of breast lesions, ranging from benign to in situ and invasive malignant tumors [1,2]. Owing to growth inside the ducts, most of the malignant papillary breast tumors are in situ, including encapsulated and solid papillary carcinoma [3,4]. The term invasive papillary carcinoma (IPC) is reserved for rare invasive breast tumors showing greater than 90% papillary morphology. Applying the above-mentioned morphological criteria, the incidence of IPC is rare (0.5% of all breast cancers) [5]. It is noteworthy that invasive carcinoma arising in the background of solid and encapsulated papillary carcinoma is not considered IPC. Moreover, invasive micropapillary carcinoma (IMPC) is also excluded from the category of IPC, as IMPC is characterized by nests of tumor cells with reverse polarization, present in cleft-like spaces. IMPC is associated with frequent lymphovascular invasion and poor prognosis [6]. Furthermore, the diagnostic consideration includes metastasis from other sites, notably, lung and female genital tract, especially, ovary. The clinical, epidemiological and pathological characteristics of IPC are not widely described in the existing literature; therefore, in this study, we evaluated the clinicopathological features and biomarker profile of IPC and compared it with invasive ductal carcinoma (IDC) diagnosed in the same study duration.

## Materials and methods

A retrospective study was conducted in the Department of Histopathology, Liaquat National Hospital and Medical College, from January 2013 to December 2020. Cases with a biopsy-proven diagnosis of primary breast cancer, undergoing excision were included in the study. Cases with metastasis or those who received neo-adjuvant chemoradiation before surgery were excluded from the study. Specimens included modified radical mastectomy (MRM), simple mastectomy with sentinel lymph node sampling (intraoperatively), and wide local excision with/without axillary dissection. All specimens were received in the histopathology laboratory and were grossly examined, followed by gross dissection according to standard protocols. Representative sections were taken from tumor, resection margins, skin (for mastectomies), non-neoplastic breast tissue, and axillary lymph nodes. During the study period, 44 cases of IPC and 1,268 cases of IDC were diagnosed. Slides and blocks of all cases were retrieved and histopathological diagnosis was reviewed. Estrogen receptor (ER), progesterone receptor (PR), human epidermal growth factor receptor 2 (HER2/neu), and Ki67 immunohistochemical (IHC) stains were applied on representative tissue blocks. More than 1% expression ER and PR was taken as positive. Nuclear staining for Ki67 was interpreted in the hot spots (area of the tumor with maximum staining) and reported as an average percentage of staining. For HER2neu IHC interpretation, complete strong staining in more than 10% of tumor cells was taken as positive (3+). An equivocal (2+) HER2/neu IHC result was further tested by fluorescence in situ hybridization (FISH) study for HER2/neu gene amplification.

Data analysis was performed using Statistical Package for Social Sciences (Version 26.0, IBM Inc., Armonk, USA). Independent t-test, Chi-square, and Fisher’s exact tests were used to check the association. P-values < 0.05 were considered significant.

## Results

The mean age of the patients with IPC was 58.77±8.38 years, and most of the patients were above 50 years of age. The mean Ki67 index was 19.95±21.12%, and most patients (59.1%) had Ki67 index less than 15%. The mean tumor size was 32.41±17.39mm, and most tumors (59.1%) were tumor (T)-stage T2. Axillary metastasis was present in 13.6% cases, and 86.4% cases had nodal (N)-stage N0. Most of the tumors were grade II (59.1%). ER and PR expression was noted in 72.7% cases, and HER2/neu positivity was seen in 13.6% cases. The lymphovascular and dermal lymphatic invasions were seen in 4.5% and 9.1% cases, respectively (Table 1).

**Table 1:** Clinicopathologic characteristics of invasive papillary carcinoma (n=44) SD, standard deviation; T, tumor; N, nodal; ER, estrogen receptor; PR, progesterone receptor; HER2/neu, human epidermal growth factor receptor 2

Clinicopathologic characteristics	Values
Age (years), mean±SD	58.77±8.38
Age groups	
31-50 years, n (%)	10 (22.7)
51-70 years, n (%)	30 (68.2)
>70 years, n (%)	4 (9.1)
Ki67 index (%), mean±SD	19.95±21.12
Ki67 index groups	
<15%, n (%)	26 (59.1)
15%-24%, n (%)	8 (18.2)
25%-44%, n (%)	8 (18.2)
>44%, n (%)	2 (4.5)
Tumor size (mm), mean±SD	32.41±17.39
T-stage	
T1, n (%)	14 (31.8)
T2, n (%)	26 (59.1)
T3, n (%)	4 (9.1)
Axillary metastasis	
Present, n (%)	6 (13.6)
Absent, n (%)	38 (86.4)
N-stage, n (%)	
N0, n (%)	38 (86.4)
N1, n (%)	2 (4.5)
N2, n (%)	2 (4.5)
N3, n (%)	2 (4.5)
Tumor grade	
Grade I, n (%)	4 (9.1)
Grade II, n (%)	26 (59.1)
Grade III, n (%)	14 (31.8)
Laterality	
Left, n (%)	18 (40.9)
Right, n (%)	26 (59.1)
Specimen type	
Modified radical mastectomy, n (%)	14 (31.8)
Simple mastectomy with sentinel lymph node dissection, n (%)	12 (27.3)
Wide local excision with/without axillary dissection, n (%)	18 (40.9)
ER	
Positive, n (%)	32 (72.7)
Negative, n (%)	12 (27.3)
PR	
Positive, n (%)	32 (72.7)
Negative, n (%)	12 (27.3)
HER2/neu	
Positive, n (%)	6 (13.6)
Negative, n (%)	38 (86.4)
Lymphovascular invasion	
Present, n (%)	2 (4.5)
Absent, n (%)	42 (95.5)
Dermal lymphatic invasion	
Present, n (%)	4 (9.1)
Absent, n (%)	42 (90.9)

Table 2 compares the pathological characteristics of IPC with IDC. A significant difference in clinicopathological characteristics was noted between IPC and IDC, with respect to age, Ki67 index, T-stage, N-stage, lymphovascular invasion, PR and HER2/neu expression. IPC cases had a higher mean age than IDC. Conversely, IPC had a lower mean Ki67 index than IDC. Similarly, IPC cases were found to have a lower frequency of axillary metastasis than IDC. IPC was noted to have a lower frequency of T3-stage and lymphovascular invasion than IDC. A higher expression of PR and lower frequency of HER2/neu expression were noted in IPC than IDC. Although a higher frequency of IPC showed ER expression (than IDC), the difference was not statistically significant.

**Table 2:** Comparison of clinicopathologic characteristics of invasive papillary carcinoma with invasive ductal carcinoma of breast *Independent t-test was applied, **Chi-square test was applied, ***Fisher’s exact test was applied, ****p-value significant as <0.05 SD, standard deviation; T, tumor; N, nodal; ER, estrogen receptor; PR, progesterone receptor; HER2/neu, human epidermal growth factor receptor 2

Clinicopathological characteristics and immunohistochemical expression	Values		P-value
	Invasive ductal carcinoma (n=1268)	Invasive papillary carcinoma (n=44)	
Age (years)*, mean±SD	51.95±12.15	58.77±8.38	<0.0001****
Ki67 index (%)*, mean±SD	30.54±21.60	19.95±21.12	0.001****
Ki67 index groups**			
<15 %, n (%)	362 (28.5)	26 (59.1)	<0.0001****
15%-24%, n (%)	286 (22.6)	8 (18.2)	
25%-44%, n (%)	282 (22.2)	4 (9.1)	
>44%, n (%)	338 (26.7)	6 (13.6)	
Tumor size (mm)*, mean±SD	36.11±14.84	32.41±17.39	0.708
T-stage**			
T1, n (%)	166 (13.1)	16 (36.4)	<0.0001****
T2, n (%)	906 (71.5)	24 (54.5)	
T3, n (%)	196 (15.5)	4 (9.1)	
Axillary metastasis**			
Present, n (%)	636 (50.2)	6 (13.6)	<0.0001****
Absent, n (%)	632 (49.8)	38 (86.4)	
N-stage**			
N0, n (%)	640 (50.5)	38 (86.4)	<0.0001****
N1, n (%)	260 (20.5)	2 (4.5)	
N2, n (%)	170 (13.4)	2 (4.5)	
N3, n (%)	198 (15.6)	2 (4.5)	
Tumor grade***			
Grade I, n (%)	106 (8.4)	4( 9.1)	0.180
Grade II, n (%)	586 (46.2)	26 (59.1)	
Grade III, n (%)	576 (45.4)	14 (31.8)	
Laterality**			
Left, n (%)	630 (49.7)	18 (40.9)	0.252
Right, n (%)	638 (50.3)	26 (59.1)	
ER**			
Positive, n (%)	798 (62.9)	32 (72.7)	0.185
Negative, n (%)	470 (37.1)	12 (27.3)	
PR**			
Positive, n (%)	646 (50.9)	32 (72.7)	0.004****
Negative, n (%)	622 (49.1)	12 (27.3)	
HER2/neu**			
Positive, n (%)	446 (35.2)	6 (13.6)	0.003****
Negative, n (%)	822 (64.8)	38 (86.4)	
Lymphovascular invasion**			
Present, n (%)	314 (24.8)	2 (4.5)	0.002****
Absent, n (%)	954 (75.2)	42 (95.5)	
Dermal lymphatic invasion**			
Present, n (%)	156 (12.3)	4 (9.1)	0.522
Absent, n (%)	1112 (87.7)	40 (90.9)	

## Discussion

We found a low frequency of IPC in our population. Conversely, IPC cases were noted to have a better pathological profile, in terms of prognostic features, such as lower Ki67 index, T-stage and N-stage than IDC. Similarly, a higher frequency of PR and lower frequency of HER2/neu expression portend a better biomarker profile in IPC.

Papillary lesions of the breast encompass a range of benign and malignant lesions, ranging from intraductal papilloma, atypical hyperplasia involving a ductal papilloma, as well as ductal carcinoma in situ, solid papillary carcinoma, encapsulated papillary carcinoma and IPC [1,2]. The incidence of IPC is low compared to IDC [5], and the prognosis is better than other malignant entities of the breast [2,5].

The differential diagnoses include solid papillary carcinoma, encapsulated (intra-cystic) papillary carcinoma, IMPC and metastatic papillary carcinoma [2,5]. The typical age at presentation of IPC ranges from 63 to 67 years and presenting complaints include bloody discharge from the nipple, an abnormal mass, and radiographic abnormalities. IPC can arise in a preexisting papilloma, but it is not always the case. Metastasis is rare in IPC. IPC is usually positive for ER and gross cystic disease fluid protein-15 (GCDFP-15) with variable-positive staining for synaptophysin and neuron-specific enolase. Myoepithelial markers such as calponin, smooth muscle myosin heavy chain, and tumor protein-63 (p63) are negative in the peripheral cells and the cells surrounding papillary cores [1,2,7].

Vural et al. analyzed data of 24 cases of IPC and found that IPC had an overall better survival and prognosis than other breast cancers [8]. IPCs are usually unilateral but can have a bilateral presentation rarely [9].

We acknowledge a few limitations of our study. Most importantly, clinical follow-up data were not available to compare the difference in cancer-specific and overall survival between IPC and IDC. Moreover, the number of cases of IPC was low.

## Conclusions

IPC is a rare variant of invasive breast carcinoma. It is important to distinguish IPC from in situ variants of papillary carcinoma, as well as IDC. We found a prognostically better pathological and biomarker profile of IPC than IDC. Apart from the lower frequency of lymphovascular invasion and axillary metastasis, and lower T-stage and Ki67 index, IPC also had a higher frequency of PR and lower frequency of HER2/neu expression. All these features confer a favorable prognostic and biomarker profile of IPC; however, large-scale studies are needed to compare survival differences between IPC and IDC.
